# The Impact of Titanium Hydroxyapatite Doping on the Mechanical and Biological Properties of Photocured Resin

**DOI:** 10.3390/mi15081040

**Published:** 2024-08-16

**Authors:** Xiaopan Li, Chao Yao, Junfu Shen, Siqi Zhu, Yiyun Kong, Chun Yao, Yuankai Zhou, Jing Xia

**Affiliations:** 1 College of Mechanical Engineering, Jiangsu University of Science and Technology, Zhenjiang 212000, Chinazhouyuankai@just.edu.cn (Y.Z.); 2 Department of Oral and Maxillofacial Surgery, Jinan Stamotological Hospital, Jinan 250001, China; 3 Department of Stomatology, Zhenjiang First People’s Hospital, People’s Hospital Affiliated to Jiangsu University, Zhenjiang 212000, China; 6000000500@ujs.edu.cn

**Keywords:** photocured composite resin, doping elements, mechanical properties, biological performance, hydroxyapatite

## Abstract

Photocured resin materials are widely used in various fields, such as 3D printing, medical applications, and dentistry. However, the strength, wear resistance, and antibacterial properties of photocured resin are relatively limited, rendering it susceptible to potential failures. In this recent study, photocured composite resins incorporating titanium-doped hydroxyapatite (Ti-HAp) were fabricated to investigate their mechanical and biological properties. It was found that the hardness and wear resistance increased with the addition of an appropriate amount of hydroxyapatite (HAp). Specifically, the 6wt%HAp resin demonstrated superior hardness. Compared with the 6wt%HAp resin, the acid resistance and wear resistance improved when an appropriate amount of Ti-HAp was added. Notably, the resin containing 0.56%Ti-HAp demonstrated superior wear resistance. Additionally, the antibacterial performance improved with higher titanium (Ti) content, showcasing a 71.9% improvement in the resin containing 1.37%Ti-HAp compared with the 6wt%HAp resin, alongside commendable remineralization capabilities. In summary, the Ti-HAp composite resin showed enhanced mechanical and biological properties, meeting clinical standards in terms of mechanical and antibacterial properties.

## 1. Introduction

Photocurable resin materials can solidify into strong and rigid objects when exposed to light. They have several advantages, such as rapid curing, high precision, and accurate control. Consequently, they are extensively utilized in three-dimensional (3D) printing, photolithography, construction, medicine, dentistry, etc. [1]. In recent decades, there have been significant advancements in research on photocurable resin materials. Researchers are working on developing highly efficient and application-specific photocurable resin materials to meet the increasing demand. In the field of 3D printing, the use of high-strength and high-precision resin materials is crucial for creating intricate structures. In photolithography, resin materials with lower viscosity and higher photosensitivity are necessary to achieve improved resolution and speed [2]. In dentistry, resin materials must have excellent mechanical properties, wear resistance, and antibacterial features to ensure long-term durability. Photocured resin typically consists of an organic resin matrix and inorganic fillers. Among these components, the type and structure of the inorganic filler significantly impact the mechanical properties of the composite resin [3]. One of the most effective methods for enhancing resin performance is to optimize its formulation through synthesizing composite resins with varying functionalities by adjusting the composition components to meet specific application requirements [4].

The main inorganic component of human teeth and bones is hydroxyapatite (Ca_10_(PO_4_)_6_(OH)_2_, HAp) [5]. HAp demonstrates excellent biocompatibility and osteoinductivity [6], making it a highly sought-after inorganic filler due to its non-toxic and harmless nature in the human body. As a result, HAp is widely used in oral and orthopedic applications [7]. However, HAp has drawbacks such as inadequate mechanical properties and brittleness, which significantly impede its progress. Currently, research is focused on modifying HAp, primarily through the synthesis of composite materials and ion doping, in order to optimize its overall performance [8,9]. Experimental researchers have demonstrated the substitution or doping of various metal ions into HAp, thereby imparting the characteristics associated with these metal ions [10]. Sebastiamml et al. [11] synthesized Ag^+^-doped HAp with cubic and rod-like structures using the sol–gel method. They investigated its antibacterial properties and cytotoxicity and discovered that Ag-Hap nanopowders containing turmeric extract exhibited potent inhibitory effects against Escherichia coli and Pseudomonas aeruginosa, demonstrating significant antibacterial activity. Bazin et al. [12] created copper-doped HAp by facilitating a high-temperature solid-phase reaction using a mixture of HAp and copper oxide (CuO) powder. They found that the presence of copper did not have a significant impact on cell proliferation, though there was a slight reduction in cell activity. However, the overall activity remained above the cell toxicity threshold established by the International Standard Organization. De Lima et al. [13] employed the co-precipitation method to synthesize zinc-doped hydroxyapatite (Zn-HAp) nanopowders using a phosphopeptide as a template for binding Zn^+^ with HAp. They observed that Zn-HAp exhibited potent inhibitory effects against both *Gram-positive* and *Gram-negative* bacteria. Ahmed et al. [14] prepared gold- and carbonate-doped hydroxyapatite (Au-C-HAp) coatings on alumina surfaces using pulsed laser deposition. This study found that as the amount of Au^+^ increased, the shear modulus of the coating initially increased, and then decreased, while the elastic modulus gradually decreased. However, the microhardness varied from 31 to 34 GPa, suggesting that the addition of Au^+^ can improve the mechanical properties of HAp coatings. Zhou et al. [15] employed the hydrothermal method to fabricate a Zn-HAp coating on the surface of ZK60 magnesium alloy. The investigation revealed that the incorporation of zinc altered the morphology of the coating, resulting in a nanocrystalline whisker structure. It was observed that this implant exhibited favorable osteoinductive capability, antibacterial activity, and corrosion resistance.

In recent years, researchers have extensively studied the impact of doping inorganic fillers on the performance of photocurable resins. Hong et al. [16] incorporated zirconia oxide into a composite resin, and their findings demonstrated that the addition of a nanopowder zirconia filler significantly enhanced the mechanical properties of the resin composite material. Li et al. [17] mixed Zn-doped HAp (Zn-HAp) and Sr-doped HAp (Sr-HAp) with a resin matrix that contained barium glass powder. They conducted experiments to study the inhibitory effects of these mixtures on Staphylococcus aureus and their ability to promote remineralization in simulated body fluid. The results showed that the composite resin incorporating Zn-HAp and Sr-HAp displayed improved biomineralization and antibacterial performances. Wang et al. [18] conducted a study on how the concentration of camphorquinone (CQ) affects the mechanical properties and curing depth of photocured dental resin. They varied the concentration of the photoinitiator CQ and found that increasing the CQ concentration below 0.5wt% improved the conversion rate and mechanical properties, such as the modulus of elasticity and hardness. Chen et al. [19] incorporated methacrylate-based resin matrices with silica (SiO_2_) particles to create MA-POSS (methacrylate-polyhedral oligomeric silsesquioxane)/SiO_2_ hybrid particles. These hybrid particles were added as reinforcing agents to a photocurable composite resin, leading to significant enhancements in bending strength, the bending modulus, compressive strength, and fracture toughness of the composite material. Sodagar et al. [20] incorporated nanopowder titanium dioxide (TiO_2_) into a composite resin and studied the effects on both the antibacterial properties and shear strength. Their findings demonstrated that adding TiO_2_ particles improved the antibacterial performance. However, this improvement was accompanied by a decrease in average shear strength. Chen et al. [21] modified the surface of sea urchin-shaped serrated hydroxyapatite (USHA) to obtain silicified USHA particles using tetraethyl orthosilicate as a silica source (TUSHA). These modified particles were used as fillers with varying loading fractions to strengthen the dental composite materials, resulting in exceptional flexural strength.

The current study has mainly focused on improving the individual performance of resin, such as compressive and bending strength, as well as antibacterial properties. However, the composite performance of resin materials has not been studied enough. This study aims to investigate a new composite resin with both excellent mechanical and biological properties. In this study, the titanium-doped HAp (Ti-HAp) fillers were prepared using a combination of the sol–gel method and sol–gel impregnation method. These fillers replaced conventional HAp in a photocurable resin matrix. The incorporation of Ti-HAp resulted in improved wear resistance and acid resistance, along with demonstrated antibacterial properties, while retaining HAp’s remineralization properties. These findings provide a theoretical foundation for advancing the development and application of photocurable resin materials.

## 2. Experimental Section

### 2.1. Ti-HAp Nanopowder Sample Preparation

Ti-HAp nanopowder samples were prepared using the sol–gel method. First, 2 mL of tetrabutyl titanate was dissolved in 50 mL of deionized water (H_2_O) (chemical Equation (1)). Then, 10.4 mL of 50% hydrogen peroxide was added. The mixture was sonicated for 10 min, and 1 mL of acetylacetone was added before heating the solution at 90 °C for 20 min to create a stable anatase titanium dioxide aqueous sol with a concentration of 3.26% [22]. Next, the HAp was soaked in the diluted titanium dioxide (TiO_2_) aqueous sol for 4 h. The white product obtained was filtered, dried, and then ground for 5 min using a planetary ball mill and sieved through a 400-mesh screen. After being calcined at 600 °C for 40 min, it was transferred to room temperature and allowed to stand for 2 h to cool down, resulting in the formation of Ti-HAp (chemical Equation (2)). The experimentally synthesized Ti-HAp is denoted by Ca_10−x_Ti_x_(PO_4_)_6_(OH)_2_, where x represents the amount of incorporated titanium (Ti). To create Ti-HAp samples with different levels of Ti doping, the amount of TiO_2_ aqueous sol solution was varied, while keeping the reaction time, temperature, and speed constant (as shown in Table 1). The dispersion of nanopowders in resin was enhanced by modifying the prepared HAp and Ti-HAp powders with a silane coupling agent KH570.

Ti(OC_4_H_9_)_4_ + 4H_2_O → TiO_2_ + 4C_4_H_9_OH (1)

Ti^4+^ + Ca_10_(PO_4_)_6_(OH)_2_ → Ca_10−x_Ti_x_(PO_4_)_6_(OH)_2_ + Ca^2+^ (2)

### 2.2. Preparation of the Ti-HAp Photocured Composite Resin

**Figure 1:**
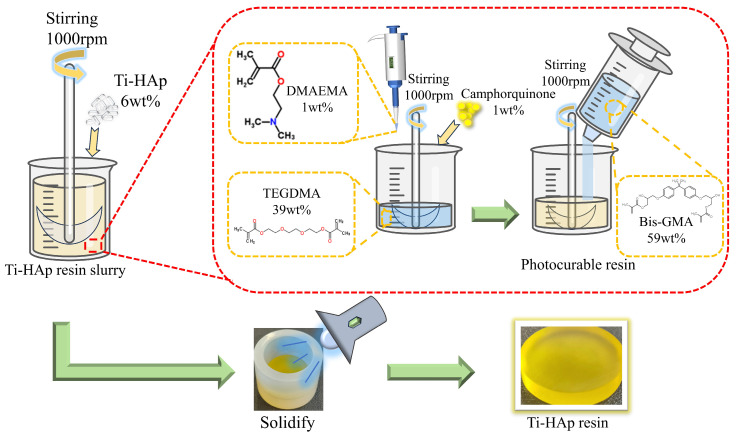
The procedure for preparing titanium-doped hydroxyapatite (Ti-HAp) resin.

HAp was incorporated into a mixture of triethylene glycol dimethacrylate (TEGDMA) and bisphenol A-glycidyl methacrylate (Bis-GMA) in a mass ratio of 4:6, and then vigorously stirred to achieve uniform dispersion. Then, CQ (initiator) and 2-dimethylaminoethyl methacrylate (DMAEMA) (co-initiator) were added in mass ratios of 1.0wt% each, followed by continuous stirring. After thorough mixing, the initiator system was placed in a dark thermostat at 40 °C for 1 h to remove the bubbles. Finally, the mixture was cured using a specialized lamp (λ = 420 nm) for 60 s to produce the HAp photocured composite resin with dimensions of Φ25 mm × 6 mm (depicted in Figure 1). The method for preparing Ti-Hap-doped composite resin is similar, with different amounts of Ti-HAp added to ensure distinct doping levels (the data are shown in Table 2). The resulting resin samples were polished using sandpaper with grit sizes of 600, 1200, and 2000, in turn, followed by polishing with 0.5 µm diamond paste on a rotary polishing machine with constant water irrigation.

### 2.3. Characterization of Ti-HAp Nanopowders

The morphology of the Ti-HAp powder was analyzed using a field emission scanning electron microscope (SEM) (Regulus-8100, Hitachi, Tokyo, Japan), and the element distribution was observed using an energy-dispersive spectrometer (EDS). The Ti and calcium (Ca) elements contents in the various Ti-HAp powders were quantified by the inductively coupled plasma (ICP) analysis using an inductively coupled plasma spectrometer (ICAP-7200, Thermo Fisher, Waltham, MA, USA). Based on the detection results, the quantities of Ti and calcium elements were calculated, and their molar ratio was then determined.

### 2.4. Mechanical Capacity of Ti-HAp Photocured Composite Resin

The hardness of the resin samples was measured using a Vickers microhardness tester (HVS-1000XAT, Grows Instrument, Shanghai, China). A 50 gf load was applied to the indenter, and 30 measurements were taken from each sample. Three replicates were tested for each group, and the average hardness value was calculated. To assess the resistance to acid etching, the changes in hardness before and after exposure to acid were compared. The test sample was divided into 4 groups, with 3 parallel samples in each group being immersed in 50 mL of a 0.004 mol/L citric acid solution at a low speed for 30 min. The remaining acid on the resin surface was washed off with distilled water, and then hardness was measured after air drying, to find the average value.

The tribological properties of the sample were evaluated using a multi-functional friction and wear tester (MFT-5000, Rtec, SAN Jose, CA, USA). A 316 L stainless steel counter-ball of 4 mm in diameter was chosen for a friction test. The friction test was carried out with a normal load of 5 N, a contact frequency of 2 Hz, a displacement amplitude of 1 mm, for a duration of 34 min, and for 4080 cycles.

### 2.5. Biological Properties of Ti-HAp Photocured Composite Resin

The flat coating method was employed to evaluate the antibacterial efficacy of the composite resin against *Streptococcus mutans* (Shanghai Conservation Biotechnology Center, Shanghai, China). The samples were placed in 24-well plates and divided into 6 groups, each with 3 replicates. The concentration of the bacterial suspension was adjusted to meet the No. 2 McFarland standard before use. Then, 3 consecutive dilutions of the bacterial solution were performed, with each successive dilution being 10 times less concentrated than the previous one. As a result, the final diluted solution was 1000 times less concentrated than the original. The bacterial concentration is 6 × 10^5^ CFU/mL. Finally, 1 mL of the bacterial solution was added to each well. After exposure to visible light at a wavelength of 365 nm for 30 min, 100 µL of the bacterial suspension was extracted, diluted 100-fold with phosphate-buffered saline (PBS: Na_2_HPO_4_ (8 mM), NaCl (136 mM), KH_2_PO_4_ (2 mM), KCL (2.6 mM), pH 7.0) and evenly spread onto the agar plates in droplets of approximately 100 µL each. After incubating the cultures at 37 °C for 24 h in a constant temperature incubator, the average colony count was determined for each group using a colony counter. The formula for calculating antibacterial rate is as follows:

R = ((βcontrol − βsample)/βcontrol) × 100%(3)

Among them, βcontrol represents the bacterial count in the control group, while βsample represents the bacterial count in the experimental group.

For the remineralization test, the resin samples were soaked in 10 mL of artificial saliva (NaCl (0.4 g), KCl (0.4 g), NaH_2_PO_4_·2H_2_O (0.78 g), CaCl_2_·2H_2_O (0.795 g), Na_2_S·2H_2_O (0.005 g), urea (1.0 g), H_2_O (1 L), pH 7.0) at 37 °C for 168 h, with the artificial saliva being replaced every 24 h. The remineralization performance was evaluated using a laser confocal microscope (LCSM, VK-X1000, Keyence, Osaka, Japan).

### 2.6. Statistical Analysis

The statistical software SPSS25 (International Business Machines Corporation, Armonk, NY, USA) was utilized to conduct a test aimed at identifying any significant differences between the samples. A minimum of 3 parallel samples were employed for each group. If *p* < 0.05, it signifies a difference between the samples, denoted by an asterisk (*). If *p* < 0.01, it indicates a significant difference between the samples, denoted by double asterisks (**).

## 3. Results and Discussion

### 3.1. Characterization of Ti-HAp Powder

The Ti and Ca element contents were quantified using inductively coupled plasma analysis. Based on the ICP analysis results presented in Table 3, the Ti/Ca molar ratio was calculated. A doping efficiency of 30% for Ti elements was observed. The ion doping efficiency is influenced by factors such as the ion radius [23]. The higher the similarity between the ionic radius of an element and that of Ca^2+^, the greater the relative doping rate. With an ionic radius of 0.073 nm, copper achieves a doping efficiency of 41.4% when combined with HAp [8]. Given that the ionic radius of Ca^2+^ (0.100 nm) is significantly larger than that of Ti^4+^ (0.060 nm), a Ti ion doping efficiency of 30% is considered typical when compared to Cu^2+^ as a reference.

**Figure 2:**
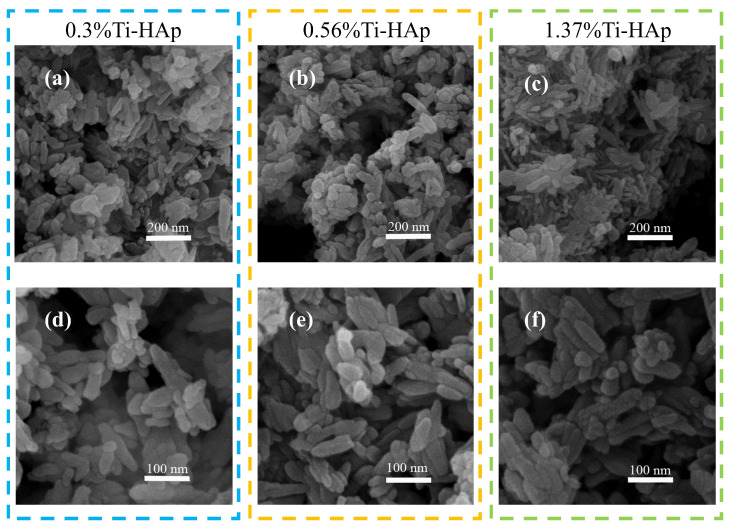
The SEM images of grain morphology of various Ti-HAp powders.(**a**–**c**) The powder morphology of 0.3%Ti-HAp, 0.56%Ti-HAp, and 1.37%Ti-HAp at 200 nm scale; (**d**–**f**) The powder morphology of 0.3%Ti-HAp, 0.56%Ti-HAp, and 1.37%Ti-HAp at 100 nm scale.

**Figure 3:**
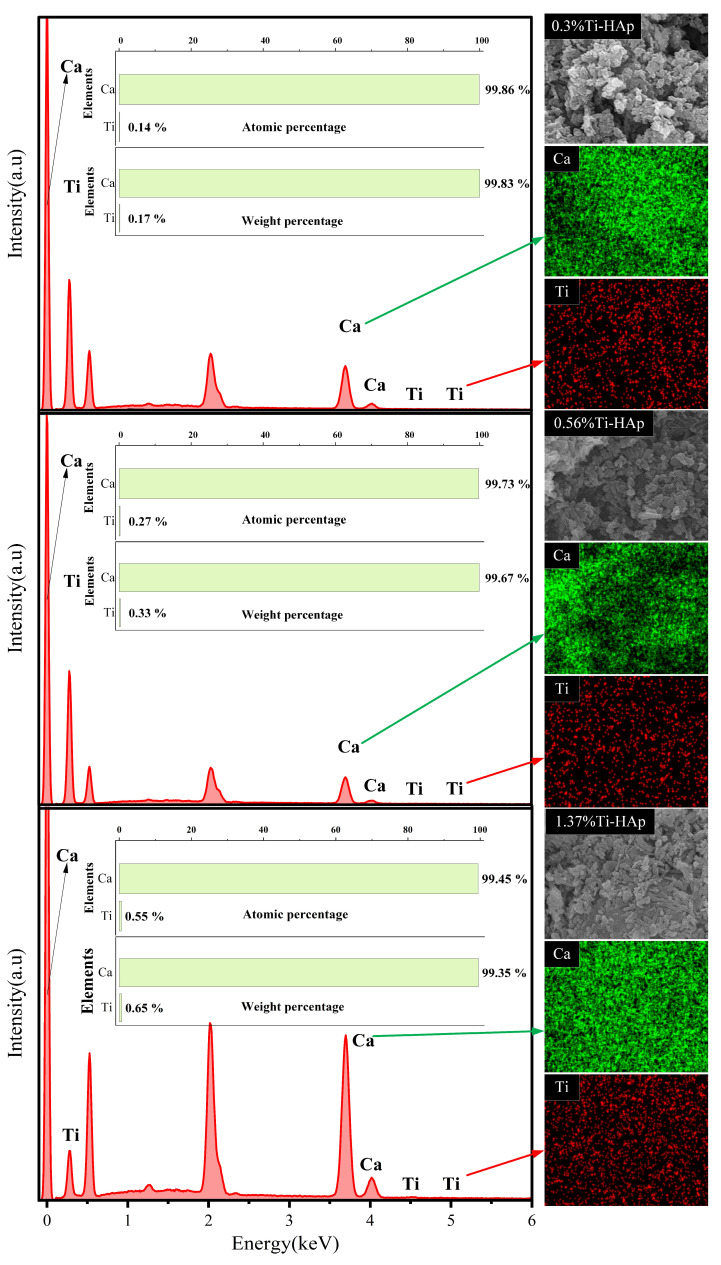
EDS mapping of various Ti-HAp powders.

The grain morphologies of the various Ti-HAp powders were examined using a scanning electron microscope (SEM). All the Ti-HAp powder samples had a typical short rod-like structure consistent with HAp [24] (depicted in Figure 2). The grain size remained in the nanometer-scale range. The combination of mapping and EDS results (depicted in Figure 3) reveals a uniform distribution of Ca and Ti elements. The Ti-HAp particles were arranged horizontally, vertically, or at various angles to the scanning surface. As a result, the grain sizes depicted in the images varied, showing inconsistent grain size. This variability makes it difficult to determine the grain size through the SEM image. By considering the atomic and mass percentages, when comparing atomic and mass percentages, the mass percentages of Ti and Ca elements in 0.3%Ti-HAp are 0.17%. When the proportion of rutile TiO_2_ aqueous solution is increased from 1.24 mL to 2.5 mL, the corresponding doped Ti-HAp mass percentage also doubles to 0.33%. When the volume of the anatase TiO_2_ aqueous solution was increased from 1.24 mL to 3.78 mL, the corresponding mass percentage of doped Ti-HAp was also increased. The mass percentages of Ti and Ca elements in 1.37%Ti-HAp were 0.65%. The concentration of Ti element increases proportionally with the augmentation of anatase TiO_2_ aqueous. The EDS findings exhibit a consistent pattern with the ICP results, providing further evidence for the successful doping of the Ti element into the HAp crystal lattice.

### 3.2. The Influence of HAp Doping on the Mechanical Characteristics of Resin

**Figure 4:**
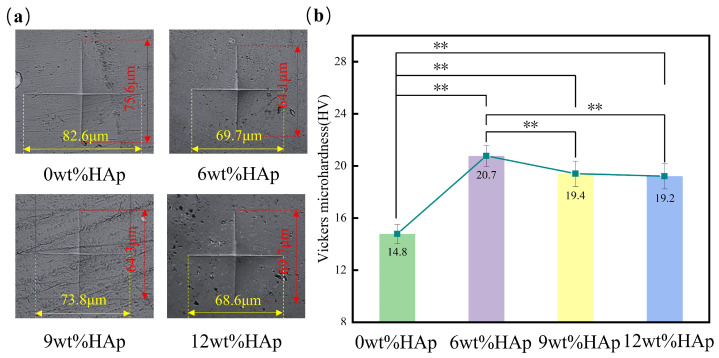
The surface hardness of hydroxyapatite (HAp) resins with different HAp powder contents. (**a**) Indentation morphology; (**b**) the mean surface hardness of HAp resins. (*n =* 3, ** *p* < 0.01.)

The hardness of the HAp resin was measured using a Vickers microhardness tester. Based on the microhardness test results shown in Figure 4a, it is clear that the size of the indentation is at its largest when the HAp content is at 0wt%, and then decreases as the HAp content increases to 6wt%. The vertical measurement decreases from 75.6 μm to 64.1 μm, while the horizontal measurement decreases from 82.6 μm to 69.7 μm. When the HAp content exceeds 6wt%, an increase in indentation size can be observed. As shown in Figure 4b, adding HAp increased the hardness of the resin. At an HAp content of 6wt%, the HAp resin had the highest hardness value of 20.7 HV, which was a significant 40% increase compared with the pure light-cured composite resin with a hardness value of 14.8 HV. However, when the HAp content was increased to 9wt%, there was a reduction in hardness of about 6.3%. Subsequently, with an increase in HAp content up to 12wt%, there was a continued decline in hardness. The increase in resin hardness that occurs when HAp nanopowder is added can be attributed to the larger specific surface area of the nanopowder. This leads to an increased contact area between the HAp and the resin matrix. When the HAp content exceeded 6wt% within the resin matrix, further incorporation resulted in the uneven dispersion and clumping of HAp particles [25], leading to poorer mechanical properties. Overall, the addition of HAp resulted in a higher hardness for the resin compared to pure light-curing composite, and this difference was statistically significant (*p* < 0.01).

**Figure 5:**
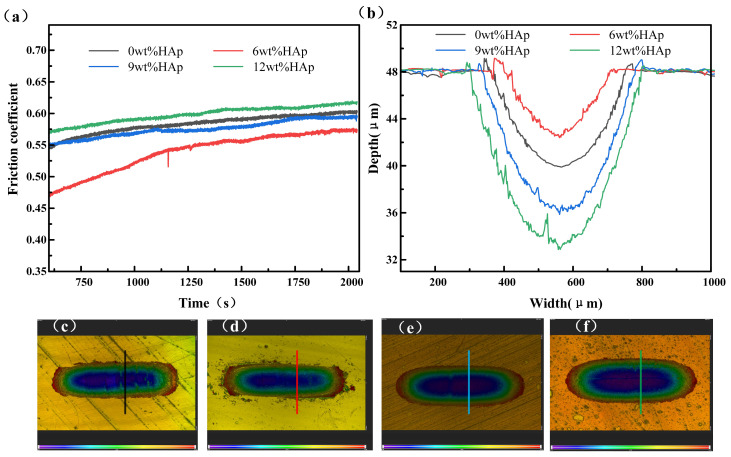
Wear performance of HAp resins containing varying amounts of HAp: (**a**) friction coefficient; (**b**) wear depth; (**c**–**f**) wear topographies of 0wt%HAp, 6wt%HAp, 9wt%HAp, and 12wt%HAp resins.

The wear resistance of the samples was evaluated using a friction and wear tester, as shown in Figure 5. The wear resistance of the composite resin initially increased with the addition of HAp content, but it then decreased. The friction coefficient of the 6wt% HAp resin stabilized at approximately 0.57 in the steady-state phase, marking a 5% reduction compared with that of the pure resin. Additionally, it exhibited minimal wear with a minimum wear depth of 5.6 μm, which was 30.8% smaller than that observed for the pure resin. The HAp nanopowder was evenly distributed within the resin at a concentration of 6wt%, helping to bear the load during the surface resin wear and peeling processes. This effectively reduced matrix wear and resulted in the lowest friction coefficient for the composite resin. As the content of HAp nanopowder increased to 9wt%, the composite resin experienced stress concentration due to the uneven dispersion of HAp, resulting in non-uniform force distribution during friction and wear [26]. Consequently, significant material detachment occurred, leading to an increase in the steady-state friction coefficient to 0.59; this approached the level observed with the pure resin. Additionally, the wear rate escalated, and the scratch depth reached 12.2 μm. A further increase in the addition amount to 12wt% resulted in both the friction coefficient and scratch depth showing further increments. The wear resistance and average hardness follow a consistent trend in accordance with the classical Archard law [27]. Typically, under constant normal load conditions, materials with lower friction coefficients exhibit superior wear resistance.

### 3.3. Effects of Ti-HAp Doping Content on Resin Mechanical Characteristics

**Figure 6:**
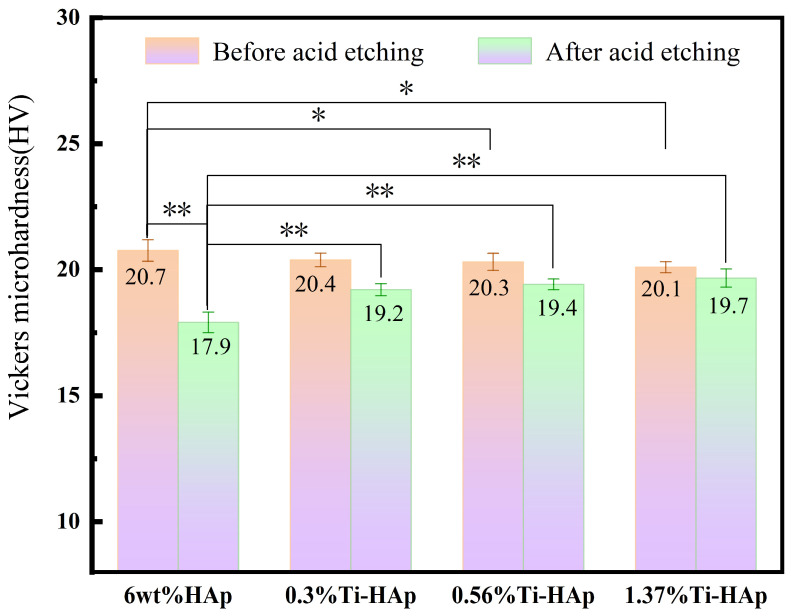
The surface hardness of Ti-HAp resins before and after acid etching. (*n =* 3, * *p* < 0.05, ** *p* < 0.01).

The Vickers microhardness of the Ti-HAp resin was assessed using a Vickers microhardness tester. The Ti-HAp resin was prepared with a consistent additive content of 6wt%. As illustrated in Figure 6, the addition of Ti had minimal impact on the microhardness of the HAp resin, but it had a different effect on the resistance to acid corrosion. After 30 min of acid etching, the surface hardness of the samples displayed a significant change, as illustrated in Figure 6. Specifically, the Vickers hardness of the 6wt% HAp sample without titanium decreased by 13.5%, dropping from 21.5 HV to 17.9 HV, a significant disparity in hardness was observed compared to the acid etching before (*p* < 0.01). The Vickers hardness of the 0.3%Ti-HAp resin decreased by 5.8% to 19.2 HV, the 0.56%Ti-HAp resin decreased by 4.4% to 19.4 HV, and the 1.37%Ti-HAp resin decreased by 2.0% to 19.7 HV. After acid etching, a significant difference in hardness decline rate was observed between the 6 wt% HAp resin and the Ti-HAp resin group (*p* < 0.01), with the Ti-HAp resin group exhibiting lower hardness decline rate than the 6wt% HAp resin. The acid resistance performance improved with the addition of Ti. In the Ti-HAp resin, the presence of Ti ions as free ions in acidic solutions is minimal, as they typically undergo a reaction with water molecules to generate a TiO_2_ thin film within the solution, as described by Equation (4) [28]. Within the etching solution, a TiO_2_ film is generated on the sample’s surface. The protective film demonstrates exceptional corrosion resistance and effectively impedes the diffusion of hydrogen and oxygen ions [29]. Therefore, the addition of Ti is associated with improved acid resistance.

Ti^4+^ + H_2_O → TiO_2_ + 4H^+^ (4)

**Figure 7:**
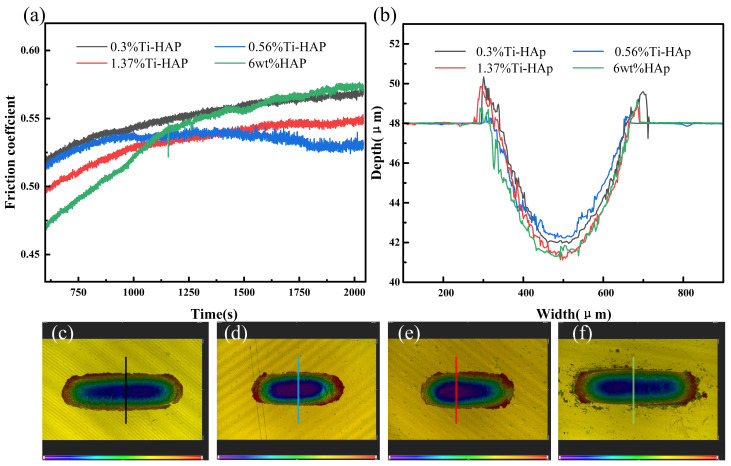
Wear performance of Ti-HAp resins: (**a**) friction coefficient; (**b**) wear depth; (**c**–**f**) wear topographies of 0.3%Ti-HAp, 0.56% Ti-HAp, 1.37%Ti-HAp, and 6wt%HAp resins.

The wear resistance of the samples was evaluated using a friction and wear tester, as depicted in Figure 7. The coefficient of friction stabilizes after about 1125 s. The 6wt%HAp resin exhibited the highest friction coefficient, while the 0.56%Ti-HAp resin showed the lowest value. When considering the wear depth, the 0.56%Ti-HAp resin shows the smallest wear depth, indicating that it has the best wear resistance. The wear resistance increases when an appropriate amount of Ti element is added. According to the literature, Ti doping effectively restricts the growth of HAp grains during sintering. The grain size of 0.8% (mass fraction) titanium-doped HAp is significantly finer than that of pure HAp [30]. The ICP analysis indicates that the titanium content in the 0.56%Ti-HAp nanopowder is 0.7% (mass fraction), which is close to the values in the literature. The finer grain size leads to a more consistent distribution of HAp particles within the photocured composite resin [31], resulting in a more uniform distribution of forces during wear. Under the wear conditions, powder not only serves as a load-bearing material but also restricts the movement of resin macromolecules, thus enhancing the wear resistance of the composite resin [32]. When the titanium content exceeds 0.56%, it leads to an increase in grain size, a reduction in the number of grain boundaries, and a decrease in the ability to impede the movement of resin molecules. Consequently, this results in an elevation of the coefficient of friction.

### 3.4. The Biological Properties of Ti-HAp Resin

**Figure 8:**
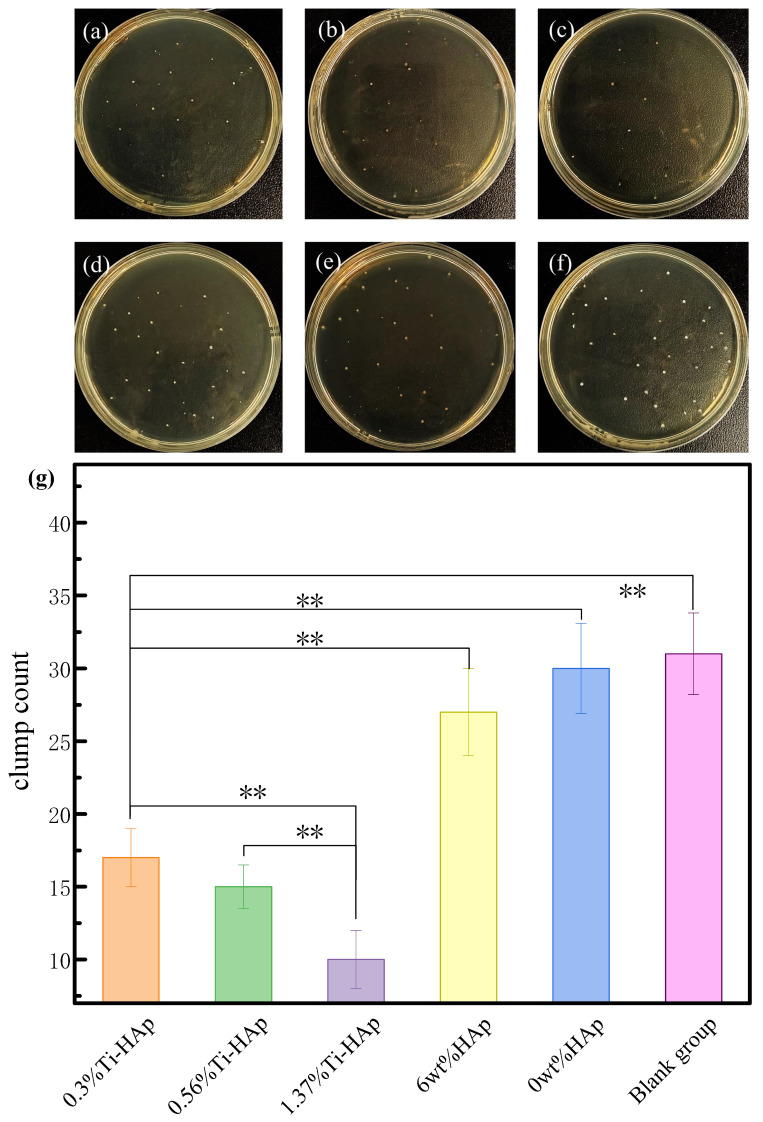
Antibacterial effect of resin surfaces under 365 nm light: (**a**) 0.3%Ti-HAp; (**b**) 0.56%Ti-HAp; (**c**) 1.37%Ti-HAp; (**d**) 6wt%HAp; (**e**) 0wt%HAp; (**f**) blank group; (**g**) average number of colonies. (*n =* 3, ** *p* < 0.01).

The exceptional antibacterial properties of resin materials not only improve their ability to prevent cavities, but also prolong their durability. The antibacterial property of photocured composite resins was analyzed through antibacterial experiments involving *S. mutans*, the main harmful bacterium that causes cavities [33]. The antibacterial effectiveness of the composite resin under 365 nm light was evaluated using the flat plate culture method, as shown in Figure 8. The 6wt%HAp resin without the presence of Ti element and the 0wt%HAp resin showed minimal antibacterial efficacy. Based on Figure 8g, the average colony counts were found to be 32 for the control group, 30 for the resin 0wt%HAp, 27 for the resin with 6wt%HAp, 17 for the resin with 0.3%Ti-HAp, 15 for the resin with 0.56%Ti-HAp, and 9 for the resin with 1.37%Ti-HAp. However, with the addition of the Ti element, the 0.3%Ti-HAp, 0.56%Ti-HAp, and 1.37%Ti-HAp resins exhibited antibacterial activities of 46.9%, 53.1%, and 71.9%, respectively. The Ti-HAp samples containing 0.3%, 0.56%, and 1.37% Ti exhibited significant differences compared to the blank group (*p* < 0.01). Ti-HAp composites that contain Ti have excellent antibacterial properties. This is because the addition of Ti improves light absorption by HAp, which leads to photoelectric reactions under ultraviolet light [34]. This process involves the creation of electron-hole pairs, which is similar to how TiO_2_ in light kills bacteria. The bactericidal effect of TiO_2_ comes from its generation of electron-hole pairs when exposed to ultraviolet radiation, producing highly oxidizing reactive oxygen species [35]. When exposed to sunlight, Ti-HAp absorbs ultraviolet light and produces highly oxidizing reactive oxygen species, which destroy the cell walls and membranes of bacteria, giving Ti-HAp its antibacterial properties.

**Figure 9:**
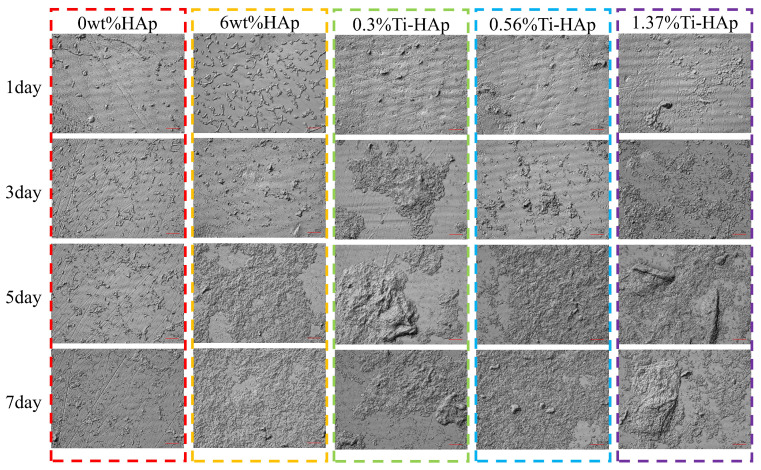
Images of the surface morphology of various resins after immersion in simulated body fluid for different durations.

Remineralization helps the photocured resin to effectively self-repair scratches and tiny cavities. The surface remineralization of each composite resin group was observed using a laser confocal microscopic morphology instrument. The surface morphology of the 0wt%HAp, 6wt%HAp, 0.3%Ti-HAp, 0.56%Ti-HAp, and 1.37%Ti-HAp resins after immersion in artificial saliva for 1, 3, 5, and 7 days is shown in Figure 9. The research findings show that the control group containing the 0wt%HAp resin exhibits a visible scratch on its surface, without any indications of mineralized deposition, indicating that non-doped resin lacks the capacity for remineralization. The 6wt%HAp, 0.3%Ti-HAp, 0.56%Ti-HAp, and 1.37%Ti-HAp resins all exhibit surface mineralization resembling hydroxyapatite. With an increasing immersion time in artificial saliva, there were increases in the compact density, deposition amount, and volume of HA-like mineralization crystals and a gradual and uniform coverage of mineralized deposits on the entire surfaces. Compared with the 6wt%HAp resin sample, the Ti-HAp group showed a significant enhancement in mineralization deposition on the surfaces of 0.3%Ti-HAp resins by the fifth day and 1.37%Ti-HAp resins by the seventh day. The surfaces of these samples show distinct mineralization nodes. The substitution of Ti ions in HAp increases the release of Ca ions and facilitates the formation of mineral nodules by providing sites for mineral impurities to nucleate [36]. The doping of Ti-HAp significantly improves the resin’s remineralization capacity, effectively repairing surface scratches and promoting the formation of mineralization nodes to facilitate the repair of tiny cavities.

## 4. Conclusions

In this paper, photocured composite resins incorporating Ti-HAp were fabricated to investigate their mechanical and biological properties. The main conclusions are as follows:

(1) With an increasing HAp content, the hardness and wear resistance of the HAp composite resin initially increased and then decreased. The composite with 6wt%HAp showed superior mechanical properties. The addition of an appropriate amount of HAp greatly improved the mechanical characteristics of the photocured composite resin.

(2) Compared with the 6wt%HAp resin, the acid resistance and wear resistance improved when an appropriate amount of Ti element was added. Notably, the resin containing 0.56%Ti-HAp demonstrated superior wear resistance due to reduced grain size.

(3) The incorporation of HAp and Ti-HAp significantly enhanced the remineralization performance of the photocured resin. Additionally, the addition of Ti-HAp greatly improved the antibacterial properties.

The research provides theoretical guidance for improving the mechanical and biological properties of photocured resin materials. The studied composite resin can be utilized in 3D printing for medical implants, surgical guides, teeth, bones, and so on. It offers enhanced antibacterial and self-healing properties, which can prolong the longevity of these applications.

In future scientific research, we can focus on the development of composite resins with improved strength, precision, and curing properties to meet the diverse requirements of photocurable resins in the field of 3D printing.

## Figures and Tables

**Table 1:** The process parameters for creating titanium-doped hydroxyapatite (Ti-HAp) nanopowder.

Ti/Ca Molar Percentage	1%	2%	3%
hydroxyapatite (HAp)	5 g	5 g	5 g
titanium dioxide (TiO_2_)	1.24 mL	2.50 mL	3.78 mL
deionized water (H_2_O)	17.297 mL	22.5 mL	18.9 mL

**Table 2:** The parameters for HAp photocured composite resin.

Pilot Sample	0wt%HAp	6wt%HAp	9wt%HAp	12wt%HAp
HAp (g)	0	0.6	0.9	1.2
Bis-GMA (g)	5.88	5.52	5.34	5.16
TEGDMA (g)	3.92	3.68	3.56	3.44
Camphorquinone (CQ) (g)	0.1	0.1	0.1	0.1
DMAEMA (g)	0.1	0.1	0.1	0.1

**Table 3:** The concentrations of calcium and titanium elements in Ti-HAp powders.

Sample Number	Sampling Quality/g	Constant Volume/mL	Element	Results		
				Conversion Content	Unit	Mohr’s Percentage
0.3%Ti-HAp	0.1025	20	Ca	375,616.98 (±1842)	mg/kg	99.7
0.56%Ti-HAp	0.1048	20	Ca	385,184.16 (±1764)	mg/kg	99.44
1.37%Ti-HAp	0.1028	20	Ca	382,608.37 (±1911)	mg/kg	98.63
0.3%Ti-HAp	0.1025	20	Ti	1426.15 (±6.2)	mg/kg	0.30
0.56%Ti-HAp	0.1048	20	Ti	2597.33 (±10.6)	mg/kg	0.56
1.37%Ti-HAp	0.1028	20	Ti	6418.29 (±17.4)	mg/kg	1.37

## Data Availability

The original contributions presented in the study are included in the article, further inquiries can be directed to the corresponding author.
